# Men´s experiences of using anabolic androgenic steroids

**DOI:** 10.1080/17482631.2021.1927490

**Published:** 2021-05-19

**Authors:** Annica Börjesson, Margaretha Ekebergh, Marja-Liisa Dahl, Lena Ekström, Mikael Lehtihet, Veronica Vicente

**Affiliations:** a Department of Laboratory Medicine, Division of Clinical Pharmacology, Karolinska Institutet, Karolinska University Hospital, Stockholm, Sweden; b Faculty of Caring Science, Worklife and Social Welfare, University of Borås, Borås, Sweden; c Department of Medicine, Karolinska Institutet, Stockholm, Sweden; d Karolinska Institutet and are working at S: t Görans hospital; e The Ambulance Medical Service in Stockholm (AISAB), Academic EMS Stockholm and Department of Clinical Science and Education, Södersjukhuset Karolinska Institutet, Stockholm, Sweden

**Keywords:** Anabolic androgenic steroids, AAS, doping, phenomenology, reflective lifeworld research

## Abstract

**Purpose**: Anabolic androgenic steroids (AAS) are used by men for their aesthetic and performance-enhancing effects and are associated with risk for side effects. Our research aims to deepen knowledge and understanding of men´s experiences of using AAS.

**Method**: This phenomenological study is based on the reflective lifeworld research approach. Lifeworld interviews were conducted with twelve men about their experiences of using AAS.

**Results**: By using AAS, men strive towards a muscular, strong and athletic ideal. Self-imposed demands, self-discipline and performance accelerate male physical development. The perfect male body ideal thus attained is fragile from both an existential and a biological perspective. The perfect self-image can easily be shattered by adversity. A man’s very existence may be jeopardized if the use of AAS is revealed to others or if the body is let down by illness.

**Conclusions**: Men´s use of AAS is a complex phenomenon. It partly concerns a traditional view of masculinity that is reflected in the community. It requires both broad and deep knowledge and understanding to be able to meet men using AAS in their problems and vulnerability; a meeting that is hampered by their low trust in healthcare, and by the fact that AAS are illegal.

## Introduction

Anabolic androgenic steroids (AAS) are considered to be a health problem because of adverse effects and their widely spread use in many countries (Eklof et al., 2003; Kanayama et al., 2018). AAS are used in supraphysiological doses (Ip et al., 2011; Lood et al., 2012) to attain the anabolic effect (Brower et al., 1994; Petersson et al., 2010) and are hugely effective (Bhasin et al., 1996; Rogerson et al., 2007). Most of the users are males who define themselves as recreational exercisers or bodybuilders (Eklof et al., 2003; Ip et al., 2011; Kanayama et al., 2020). In many parts of the world, men experience dissatisfaction with their bodies (Kelley et al., 2010) which may be a reason for using AAS (Mitchell et al., 2017; Pope et al., 1997). The main motives for using AAS are improvement of body image and appearance or enhancement of performance (Börjesson et al., 2016; Melia et al., 1996; Petersson et al., 2010). Further reported motives are low self-esteem, curiosity, desire for increased braveness or criminality (Bonnecaze et al., 2020; Ip et al., 2011; Nilsson et al., 2001).

Use of AAS can cause side effects, some are mild while others may be more serious. The risks for side effects increase with higher doses and longer duration (Bolding et al., 2002; Evans, 2004). Typical AAS-induced physical side effects in men include sexual problems most commonly erectile dysfunction and decreased libido, acne and gynaecomastia. An association has been seen with a variety of psychiatric complications (Börjesson et al., 2020; Eklof et al., 2003; Pope & Katz, 1994; Pope et al., 2014). Typical psychiatric side effects include depression, sleep disorders and mood disturbances (Sjoqvist et al., 2008). AAS use may also increase the risk of cardiovascular complications such as hypertension, disturbances in the cholesterol profile (Chang et al., 2018; Gårevik et al., 2011). Cardiovascular consequences of AAS have received attention due to case reports of sudden or unnatural death (Darke et al., 2014). Swedish legislation forbids the use of AAS, not only the possession and distribution of AAS, but also the presence of exogenously AAS in the body.

This is the first interview study with AAS users that has practised the reflective lifeworld perspective (Dahlberg et al., 2008) (RLR) with a caring science perspective (Dahlberg, 2011). In this research, we turn to the men themselves and to their lifeworld. The study aims to deepen knowledge and the understanding of men´s experiences of using AAS and explore their lived experiences. Through the RLR approach, we aimed to reach an existential dimension that is missing in previous research. A deeper knowledge is important especially for healthcare professionals since AAS is widespread and may affect an individual´s health.

## Materials and methods

### Design

This phenomenological study is based on a reflective lifeworld research (RLR) approach as described by Dahlberg et al. (2008). The epistemology of RLR is based on phenomenology mainly described by the philosopher Edmund Husserl (1936/1970). RLR is phenomenon-orientated throughout the research process (data collection and analysis) and the phenomenon being explored and illuminated in this study is men´s use of AAS. Using a lifeworld perspective helps us to seek understanding in the lived everyday world. The lifeworld is our unique existential world as it is experienced by each human being, but it is also a common world that is shared with other people.

In order to understand the phenomenon itself, it is necessary to be aware of the natural attitude. In our daily lives, the world is perceived through our natural attitude, which means that many things are taken for granted or in other words are not reflected upon. The researcher therefore needs to keep his/her distance to the natural attitude, which is achieved through reflection. By reflection, we can raise awareness and conceptualize the lifeworld.

Openness, flexibility and bridling are important methodological principles in RLR. An open mind is achieved through bridling, i.e., having control of the whole process of understanding of the phenomenon, which involves a reflective attitude and flexibility towards the phenomenon.

Researchers need to be constantly reflective and critical throughout the research process. To be objective in a phenomenological sense, personal values, theories and other assumptions may not impede us from acquiring a new understanding of meaning (van Wijngaarden et al., 2017).

### Participants and study setting

The total number of male participants in this study was twelve, 20–65 years of age. Half of the participants were ongoing users of AAS, and half were former users. For inclusion, they had to be over 18 years of age and to understand the Swedish language. Voluntary informants were recruited in two ways: either via snowball sampling or by contact with Anti-Doping Hot-Line. Snowball sampling is a method for studying social networking structures, originally introduced by Goodman (Heckathorn, 2011). This technique is a convenient method for hard-to-reach and hidden populations. Contact with an initial person can generate new informants. The second way to recruit was via Anti-Doping Hot-Line which is an anonymous free telephone counselling service started in 1993 for people concerned or affected by their non-medical use of AAS (Eklof et al., 2003). It is managed by trained nurses and clinical pharmacologists and is localized at the Department of Clinical Pharmacology, Karolinska University Hospital in Stockholm, Sweden. Half of the informants were recruited by each method.

### Data collection

The lifeworld interviews were conducted by AB and VV in an undisturbed setting. The interviews lasted 60–90 minutes and began with a presentation of the study and its purpose. Each interview started with an opening question (Brinkmann & Kvale, 2014; Dahlberg et al., 2008) directed to the phenomenon: How is it to use anabolic androgenic steroids? Follow-up questions were asked aiming to support the informant in clarifying, deepening and explaining what was meant and to encourage detailed descriptions of the phenomenon (e.g., how do you mean, can you describe more?)

### Data analysis

The phenomenological analysis followed the guidelines for RLR (Dahlberg et al., 2008), which are openness, bridling and flexibility. During the process of analysis, the researcher must adopt an open attitude. To promote this, the researcher needs to slow down the process of understanding and not be too quick to make definite what is indefinite (Dahlberg & Dahlberg, 2003). The analysis process concentrates on the phenomenon and not on the individual. The aim is to search for meanings in all the experiences collectively.

The analysis process starts with familiarization, an open-minded repeated reading of the interview transcripts in their entirety to create a deep feeling for and understanding of the whole text. Then a movement between data follows, which means pendling between the whole (interviews) and the parts (meanings of the data) and finally reconstructing a new whole (essential meaning) (Dahlberg et al., 2008). The material is divided into smaller parts (words, sentences or longer pieces of text) in the search for meaning. From a phenomenological perspective and validity, research should be meaning-orientated (van Wijngaarden et al., 2017). Reflective questions are used in order to understand the meaning of each unit, likewise it is important to reflect carefully and not take anything for granted. Reflection and questioning are repeated until meanings that seem to be related to each other can be clustered together. Once the clusters are established, the meaning of the phenomenon slowly begins to emerge and the pattern between the clusters and the essential meaning of the phenomenon becomes visible. The analysis process results in a meaning structure that includes both essential meanings and the more varied meanings. From the most abstract essential meanings, that is the essence to the more concrete and more contextual meanings, that is the constituents. All authors have participated in all steps of the data analysis.

### Ethical considerations

Ethical approval was obtained from the Regional Ethics Committee in Stockholm (nr. 2016/1762-31/5). The informants received oral and written information about the purpose of the study before and at the time of the interview. They were informed that participation was voluntary and that they had the right to withdraw their participation at any time without explanation. They also received information about the confidentiality of the interviews before giving their written consent. Participants were offered care and support if they so desired.

## Results

The results are first presented through the essential structure of meanings, followed by the meaning constituents: *the male body ideal as a model; the perfect male identity; individual existence may be jeopardized when demands are too overpowering.*

The use of AAS aims to achieve a perfect male body ideal, which traditionally is muscular, strong, athletic and healthy. Male physical development is accelerated through the use of AAS combined with high self-imposed demands, self-discipline and performance. A prerequisite for starting to use AAS is having achieved training maturity as well as having self-control and the potential for further physical development. The body is sculpted by self-centred effort directed towards the desired body ideal. An identity is created and shaped, which over time leads to self-confidence and a positive self-image. An inseparable new self-identity stimulates respect by being impressive, prominent and dominant. The challenge is to maintain the arduous and disciplined lifestyle. However, both external and internal factors may limit the body as experienced, thus weakening the self-image. The perfect self-image is fragile and can easily be shattered by adversity. The role of the perfect male ideal is maintained in interaction with the environment and kept alive through other people’s confirmation, acceptance and integrity.

### The male body ideal as a model

The men in this study strive to create the perfect physique based on the masculinity norm, i.e., behaviours and traits that traditionally characterize and are associated with masculinity. They include physical and mental strength and self-control as well as being performance-orientated, invulnerable and confident. Through hard training and discipline, these men steer their bodies towards achieving male physical perfection. They perceive their development as a reflection of their masculine role model and their perception changes as their physical development progresses.

What drives the men in this study is that they do not want to look thin, they want to get bigger, build muscle and strengthen their masculinity. One man comments:

I was extremely fascinated by these masculine men with big arm muscles and stuff. Today, when I look back, it’s often associated with what I call authoritativeness because I thought that men who were well-muscled were self-assertive in a certain way.

The men in this study are fascinated by how fast their bodies can develop with AAS. The effect makes it easier for them to steer and control their bodies in the desired direction: *“…when we used that substance, we just laughed our way through every single training session/workout for six weeks.”*

They feel satisfied with achieving what they want but cannot settle for that since they constantly want to develop even more. There are always areas for improvement and shortcomings to work with, such as more defined and better-developed muscles. It is sometimes difficult to perceive correctly the actual size of the body. To follow their physical development and get a reliable picture of their development, they use scales, photographs and mirrors to help them. Now and then they experience not looking handsome enough, or looking small, which makes them hide their bodies in large clothes to avoid attention. However, they sometimes also become aware that their bodies have actually become overdeveloped, which feels uncomfortable, as expressed in the following statement:

I weighed 117 kg for a while, you know. And I’m 1.75 tall, so that´s quite a lot. And then I was too big for my body like, to put it one way. That’s how I saw it. Then the thoughts started to come … ’hell, should I really be doing this?’ Everything was just too tight everywhere. Yeah, my muscles were tensed and tight in my skin … that way you look a bit more grotesque. You think you look small even though you’re really as big as a fucking bull … then there’s something that is very wrong …

The body is pressured and challenged to perfection. These men always give their all in full, making great demands on themselves and doing nothing light-heartedly. They are competition-orientated, disciplined and focused, which however is not always that easy to maintain, as exemplified: *“everyone wants to be in good shape, but not many have the discipline”* or *“some people are just not able to push themselves that hard.”*

There is the explicit idea that everyone should struggle to be able to reach his goals, and the perception is that everyone should do it the hard way. The tough and hard bodybuilding culture from the 1970s–1980s is highlighted as an ideal. The ancient body ideal with clearly defined muscles and symmetrically built bodies alongside recognized bodybuilders are described as role models. Based on this notion of doing things the hard way, it is not acceptable to use AAS until full muscular potential has been reached, i.e., training maturity. Only then may AAS be used to achieve the desired goal. Training maturity means that to avoid injuries, the individual must be physically developed to take on a heavier physical load, which is important because AAS provide increased energy and encourage heavier training. Training maturity also involves being able to achieve as much as possible the natural way without having added AAS. Here, however, opinions differ between the men interviewed. Some are tempted to get on the fast track and do not see their bodies as “an ongoing handicraft project” that has to be built up slowly and laboriously, i.e., if training sessions are missed or if they have not eaten properly, they increase the dose of AAS and hope that this will compensate for any lost results. Some men express that a genuine bodybuilder should behave exemplary in terms of food and exercise. Using more AAS than needed is considered as cheating, meaning that they do not get an honest and fair chance in competitions. They made comments like: *”they don’t take things that seriously”; they haven’t understood their natural potential*”; and *”your body should be your temple.”*

The risks involved in using AAS are justified by the physical results achieved. The risk of side-effects is considered small if AAS are taken with caution and in the right way, but this requires the individual´s awareness and knowledge. The level of knowledge differs between individuals; some men are deeply familiar with the topic while others rely entirely on the “facts” that their friend has read about AAS: *“but I didn’t read anything because he had read it all, so I more or less told him that I trust you”*. The choice not to acquire any knowledge at all may also be a conscious one, because the individual is afraid of not daring to try after having read about the side effects. Despite awareness of the risks of purchasing AAS substances on the black market, these individuals are prepared to take that risk. If serious side effects do not occur, they test several different types of AAS and to a greater extent, which means that they may become less careful. They also trust that the side effects will disappear when they stop their use of AAS. However, there is also a more cautious approach to AAS in which winning competitions is not the most important part. One man describes his intake of AAS as follows:

Well, if it was only important for me to win, for me to be the biggest of all like … Well, then I’d have to take much, much more than I do today. I’ve been extremely careful for various reasons. On one hand, as I said, I do not want to feel unwell. I do not want any side effects. I do not want to risk my heart and liver and everything getting damaged. So in that way, winning is not everything.

To protect their bodies during AAS use, the men interviewed tried to counteract or prevent side effects. Dietary supplements and drugs are taken in their attempts to streamline their bodies’ defence systems to protect their livers and kidneys. Healthy food compensates for AAS intake, as one man says:

When you’re on AAS substances, you should think one step ahead and be even cleaner, even more careful, eat extra greens, as well as other nutrients; choose unsprayed food because the body is more sensitive which makes the side effects worse; there’s an increase in harmful blood fats; fatty deposits in the blood vessels increase, and so on.

It is sometimes difficult to know if the problems and discomfort perceived are really due to the intake of AAS or if they have arisen for another reason. When the realization dawns about the real risks of the substances, it can be extremely upsetting. One man describes his experience after suffering from a serious illness:

You see, I’ve been so convinced up to now that this is not unhealthy, like my blood values, you know – everything is great. My body hasn’t felt unwell at all, like when you start having problems with your heart and therefore I haven’t had any motivation to stop. Had it turned out that it was bad, then I would definitely have started thinking, but then I would have thought kind of, well then I’ll stop for a while and then I can start again. But once I got this, well, it just suddenly happened one day. So my God, this is bloody dangerous stuff. It´s not like everyone else says.

If the men are worried about side effects, they contact healthcare for help. They believe that their AAS use is a private matter and that the physician’s task is to assist people if they do not feel well. Health checks can prevent injuries and predict when medical measures need to be taken. The men believe that as long as they endure certain ailments and feel relatively well, they are justified in continuing to use AAS: *” I experienced that a lot was dose-related, that I could often create balance by using different doses.”*

### The perfect male identity

The perfect male identity is acquired through competence in bodybuilding, the physically developed body, and the masculinity achieved thereby. This identity, and the successes and the confirmation received on account of it, lead to satisfaction. The arduous lifestyle results in a status that needs to be maintained. The life histories of the men interviewed were influenced by their bodies’ physical appearance and their fear of not meeting the male norm. At an early age, they experience a fascination for muscles and for the functional body as strong and good-looking. One man reports:

It´s just that I want to be big, I want big muscles. Maybe it´s something genetic, something that has been inherited, because my dad was also like that when he was younger, he trained and lifted weights, Olympic lifting. And my uncle talks about them competing against each other to see who could lift the most and so on.

The intake of AAS sometimes begins through peer pressure between men. Peer pressure concerns the fear of falling behind in development and not achieving the same physical success as those who use AAS. It is described as a form of mutual competition about performing best, lifting the heaviest weights, achieving the fastest physical development or being the best-looking and most popular with girls: *“that was the worst thinkable … Christ, you couldn’t let your best mate beat you so totally like … so that he was the most popular.”*

The motivation to strive for a bigger body may also involve not feeling scared or small:

I saw the possibility of never being afraid again in my whole life. And the bigger you are, the harder you hit. It’s also the case that nobody dares to attack a guy who weighs 120 kg, just his muscles alone.

The men interviewed experienced self-affirmation by taking care of their bodies. This self-experienced effect is proof that their strategy is working. Their new larger bodies result in greater self-confidence and a more positive self-image as the following comments show:

When I was walking along the corridor at school, I knew that everyone was watching”; and “You feel good … when I feel my muscles, when I see myself.

This greater self-confidence results in satisfaction, which was variable on different occasions depending on daily body shape. As one man says:

The mirror is also a kind of method of assessment and all mirrors are different, as many people know, so when you are at the gym you see one thing and when you get home you are completely devastated.

The men compare themselves to men they consider as having the perfect male body and not to ordinary normally built men. Getting confirmation and/or respect for their body development is vital for their wellbeing: *“You kind of wanted to hear that you were big and looked good.”* However, attention and confirmation sometimes also take too much of their energy, which they also try to avoid. Who the confirmation came from was also significant, it being more meaningful when it came from someone they look up to or respect, as one man reports:

When you went to bodybuilding shows, that was where it mattered. When you went to a stand to buy PWO or protein or something like that, and there was a fucking giant there to sell stuff, and you know, when he pointed out my shoulders for example, ‘Jesus what fine shoulders’– good shoulders are something a lot of people have difficulty in getting, so ‘fine, thanks!’ We have something there. That´s something. He pointed something out to me and then I started ‘shit ten years ago, or eight years ago, I looked like that, you know’, and now I´m here talking to the big guys you know so things are a bit different. That confirmation is important. But ordinary people sometimes said, they sometimes said lots of things, but then I just said ‘uh, look, I´m really small’, ‘no no you are huge’, ‘no I´m really small because I’m not comparing myself with you, I’m comparing myself with someone else’ and that’s how it works.

Building one’s body provides the opportunity to demonstrate skills and value. A role that accompanies the perfect male identity is the so-called “Guru*”*. This is a person whom other people ask for his advice and knowledge. The role involves status and includes special knowledge in bodybuilding that gives an individual the competence to guide others: *“He’s the one you go to for advice on which dietary supplements to take and which chest exercise to do.”*

Men who describe themselves as having a strong self-esteem, have less need for confirmation. However, a few positive words are always welcome and a pat on the shoulder is quite sufficient. Compliments are not always necessary because the men are already fully aware of their achievement, as one man notes:

I´m more of a passive confirmation seeker, I want the confirmation when I know that I have done something good or when I look good, when I know that I have fought for my body. Then it´s fun to hear from others that well you look good, but I do not need to hear it because I have the confirmation in myself.

In order to develop and maintain an identity, time needs to be set aside for daily routines. It is crucial to prioritize and follow one’s busy food and exercise schedule to achieve set goals. There is an underlying stress in being disturbed in one’s daily routines, and what is less important in everyday life has lower priority: *“Stress from not having time to eat, stress from not having time to exercise. Nothing must come between.”*

A large and muscular body implies that people around to a larger extent accept and understand the strict lifestyle. However, this lifestyle is exposed to both positive and negative comments. The positive criticism is about bodybuilders’ dedication and fighting spirit, while the negative is about the strict diet they adopt. The social context is limited and only those who accept the men´s lifestyle are included in their social circle. It can be both time-consuming and wearing to respond to all comments. Therefore, they change their social interaction or protest violently if their life choices are criticized or questioned. Their self-confidence is strengthened by the fact that they live more carefully and take better care of themselves than other people do:

So I have called people fat when they comment, then I have answered but my God I really don’t want to look like you. Then they shut up.

Another man comments:

I often ate whole cartons of ice cream with tuna and rice, that was my way of living on Sundays. That´s what I ate. I remember very well when my sister had her birthday and that somewhere I thought that I should not go to it because it was a birthday and then there would be cake, but still I took my little cooler bag and went there with my ice cream cartons and sat and ate rice and tuna when everyone else was eating cake. My father had a huge outburst against me and was very angry and felt offended that I could do such a thing that day. He had me with my back against the wall and said how the hell can you do this today. I answered him, how the hell can you do this, how the hell can you sit and eat cake, you’re destroying your body, you’ll get fat and get at high cholesterol.

### The existence may be jeopardized when demands are too overpowering

Existence may be jeopardized if the use of AAS is revealed to others or if the body is let down by illness. Society´s attitude is judgemental, which entails a need to live with lies. Lies may be difficult but necessary to avoid being treated unfairly by other people. After the revelation of AAS use, it may be difficult to carry on one’s life as usual. Dignity may be lost with fear and anxiety as a result. One man describes his concern over what he would lose:

Everything. Myself. The lot. I’ll lose myself, or my whole identity. That was me. My steroids, my training, that was all I had. That was where I was somebody, I was myself. I knew what I was doing, I had found my place.

When demands are made, by close relationships or by society at large, to return to a normal life, these men´s freedom is restricted. This overpowering demand means that their very existence is jeopardized, and it feels difficult for them to stop using AAS: *“I did not want to quit my steroids, absolutely not. I sat and cried on my sofa, you have ruined my life.”*

It is not certain that these men would choose to put their families first. One of them describes being forced to make a choice:

But then it started to get a little unbearable so she said to me ‘you must choose between me or the steroids’ and then I said ‘what a stupid question, I choose my steroids’. Then she came after a while and said that ‘either you seek help, or you are not allowed to see the children’. And that was it, that´s really the only way to get to me. So her choosing that, it was a desperate last, last thing she did.

The men are torn between continuing and ending their use, as one man explains:*“best antidepressant ever, better self-esteem and better self-confidence”.*

It is not possible for men who have used AAS to promise that they will never start again even though they might have exposed their loved ones to discomfort, been seriously ill or served a prison sentence: *“I’m completely clean now, but if I will remain clean for the rest of my life, I cannot say”.*

The strong love for AAS despite risks and consequences together with positive memories means that they constantly need to remind themselves of why they quit. One man says:

I have to, I have to constantly remind myself. If you compare with other addicts, you know they can take out an old photo where they’ve been on drugs for ten years, you know they look like skeletons and you know that it’s so ugly that they don’t want to end up there again. Then all they have to do is to take out that photo, and that’s nice for them, because then they think oh no, hell no, don’t want to end up there again. I don’t have that. I do not have a photo like that. I look like a god if I go back and look at my pictures you know. So it´s just like hell, (laughter) so then I have to go through the negative aspects all the time in my head, I have to constantly remind myself that this was not good, it was not good at all. My children were afraid of me, I threatened my wife, no I do not want to end up there again, I do not want that.

These men need to experience side effects or to be told straight out that a certain side effect is caused by AAS before they decide stop using such substances. Intimidation propaganda is not perceived as an adequate deterrent, but they are motivated to stop if it is possible for them to identify themselves with somebody affected by the side effect in question.

The men experience their authenticity and thus identity as being called into question if their use of AAS is revealed. The performances and bodily results they have achieved are experienced as being trivialized. They were given a label that is difficult to get rid of.

The interviewees believe that people outside the world of doping are convinced that AAS causes users to become aggressive and violent and thus automatically “dangerous”, which in their minds is a wrong image. All possible events, ill health and behavioural changes that affect them will be linked to their use of AAS. The word “doped” is perceived as a negatively charged word, which for them means a feeling of being “labelled”. The mass media reinforce this image and do not take into account the feelings of those who are vulnerable. Due to this exposure, users may limit the intake or dosage of AAS, so that the muscle mass does not increase too fast and arouse suspicion.

Lying feels difficult, because it may be obvious when somebody is lying, but at the same time it can be difficult to tell the truth because then you can be judged by other people. Living a lie may be tiring and affects a person’s wellbeing, and people sometimes feel it would have been easier to be honest. To avoid being dishonest, the men interviewed tell certain selected people that they are using AAS. However, it can be difficult to be honest with those closest to them. This is partly because they wanted to protect them from the truth and partly because they do not want to be exposed to their disapproval. As one man puts it:

Yes, but our relationship would probably break up. Either I would probably try to quit, which I was not prepared to do then, or we would not have any further contact. At least that’s what I believe, because my mum, she is quite strict.

Although the men consider it important to contact a physician when they experience various ailments, they only make this contact in an emergency. They then choose not to be completely truthful about their use of AAS, partly because of their concerns about consequences since AAS use is illegal and partly because they experience being treated with a condescending and judgemental attitude. This kind of attitude leads to a low level of trust towards healthcare personnel. The men feel offended or experience powerlessness. The lack of respect makes them prefer to continue their use of AAS.

However, when they meet a “factual”, honest and non-judgemental physician, they experience strong sense of trust and security. It is easier to be honest and open to people who do not judge. They also feel that in such cases they are more receptive to information about their AAS use. The men wanted an open and trusting contact with physicians, since this creates a more permissive climate regarding the use of AAS.

If the men are suffering from a serious illness, they feel that support is important. They have good experiences of support from priests, curators and psychologists when needing to end their use of AAS due to illness. Friends also play an important role when things feel difficult and daunting. Nowadays they are happy to tell their stories to process events and to be available to support others who want to quit or are having difficulty in quitting. They really want to be supportive and act as personal contacts.

After quitting the use of AAS, it may be difficult for former users to start exercising again because the effect is not as great as with AAS. The desire to exercise is affected by the lower testosterone levels that follow a discontinued use. Even though the men have normal testosterone levels, they no longer feel vital, instead experiencing depression, decreased sexual desire and lack of wellbeing. This means a suffering for which it is difficult to find help.

## Discussion

The participants in our study used AAS because they had a wish for the perfect male body, they wanted to get bigger and gain more muscle. The use of AAS gave them faster muscle development and less body dissatisfaction. Muscle dysmorphia (MD) is a form of disorder characterized by a preoccupation with muscularity and body image (Phillips et al., 2010). Male bodybuilders report MD to a greater extent (Mitchell et al., 2017; Pope et al., 1997) than non-bodybuilders resistance trainers. It has been known for many years that men take risks and experiment with different preparations to create extraordinary bodies (Gaines & Butler, 1974; Kanayama & Pope, 2012; Klein, 1993). They focus on a strict diet, extremely heavy weight training and the use of AAS (Mitchell et al., 2017; Pope et al., 1997). The men interviewed were aware of the risks of using AAS, but their concern about side effects decreased when no serious side effects appeared. In order not to expose themselves to obvious risks they tried to protect their bodies by following a healthy diet, the intake of specific supplements or the use of lower doses of AAS. Male AAS users have been shown to neglect the side effects of AAS and short-term gains generally outweigh long-term risks (Grogan et al., 2006; Kimergård, 2014). The majority of AAS users continue their use despite side effects (Ip et al., 2011) but they are concerned about harmful effects on health (Börjesson et al., 2020; Parkinson & Evans, 2006).

The results showed that the men were equipped with self-control, in other words they were disciplined and driven by will-power. This is a necessary attribute to be able to transform and shape the body to such a great degree. The body is a time-consuming project and by having self-control they were consciously able to control their decisions and choices (Egidius, 2008). The men were constantly self-critical of their bodies’ faults and shortcomings and driven by unhealthy ideals. Perfectionism has increased in recent decades which may be due to greater demands, unrealistic expectations and more competitive environments (Curran & Hill, 2019). Perfectionistic traits, especially typical for elite athletes (Lemyre et al., 2008), may include expectations such as achieving a perfect body. But if healthy striving gives way to self-imposed demands, self-critical evaluations of achievements and concerns about negative assessments (maladaptive perfectionism) it may become unhealthy (Lundh, 2004).

Large muscles, strength, increased focus, respect and sexual virility are effects desired by the men in this study. According to Andreasson and Johansson (2016), these attributes are linked to manhood and masculinity. Bodybuilding has been described as a predominantly masculine subculture (Klein, 1993) and AAS use involves an anticipated process of transformation (Andreasson & Johansson, 2016). Within the bodybuilding subculture, belonging to the highest level of the hierarchy is the goal. According to Raewyn Connell (1995), the male body is an inevitable ingredient in the construction of masculinity.

Masculinity is built around a hegemony in which men are divided into categories, so called hegemonic masculinity. There can be more than one hegemonic masculinity within a society, and it can pertain within subgroups where the ideal differs based on context and environment. Hegemonic masculinity is closely related to patriarchy and is the ideal that men relate to and strive for, but very few identify with. The hegemonic ideal is a leading position, difficult to achieve, to which men have an ambivalent and complicated relation, and which is surrounded by values, status and power. Reaching the ultimate goal is an ideal that coincides with hegemonic masculinity (Connell, 1995). By constantly supporting this ideal, the men in this study were given the benefits of hegemonic masculinity.

These men´s feelings of inadequacy indicated that they had a low self-esteem and self-confidence. The feeling of being just one of the crowd may have created the desire to stand out and might be a sign of why the men in this study felt that they needed to acquire a perfect body and appearance. Their feelings of inadequacy might have been influenced by the fact that positive qualities such as health and success are often attributed to attractive and good-looking persons (Rennels, 2012). The men in this study wanted to be seen as people who were not frivolous or light-hearted but who were dedicated, focused, hard-working and successful.

Performance-based self-esteem (PBSE) is a concept linked to perfectionism (Hallsten et al., 2005). PBSE is based on concerns of not being good enough and the results achieved are an important measure of ability (Hallsten et al., 2005; Makower, 2018). It is a variant of low self-esteem based on the individual proving her/his human value through performance. Individuals with high PBSE are often ambitious and base their value on external factors such as success and personal status. External confirmation becomes a compensation for their lack of self-esteem (Hallsten et al., 2005).

The men interviewed here received a certain status within their own group of bodybuilders depending on how well they attained their goals. Their status was affected by how hard they worked and that they did not take the easy way of cheating by using a lot of AAS substances. For some it was important that the body should be built as an ongoing handicraft project and for others it was a matter of quickly achieving a certain result or goal that would bring respect.

Through the body we have the opportunity to create and transform our identity and self-image (Giddens & Sutton, 2013). Identity is what characterizes us and makes us unique and we use different tools to reach our ideal. The physical body becomes an instrument to work with and the boundaries have been blurred for what it is realistic to try to achieve. Individualism places the responsibility on the individual her-/himself to seek her/his own way and construct her/his own identity (Giddens & Sutton, 2013). International valuation surveys show that individualism and self-expression are highly valued in Sweden (World Value Survey, 2021).

These men´s acquired position as examples of the perfect male body ideal provided them with a social role in relation with other people. Goffman’s role theory (1959) is based on people play different social roles depending on the situation and the occasion. The role is a socially defined expectation that a person with a certain status or social position tries to live up to. That kind of acquired position is achieved by the individual her-/himself and the ideal body may be seen as a result of social processes (Giddens & Sutton, 2013). The social constructivist perspective means that we are born into a society that constantly influences us and we relate to existing norms and conceptual frameworks. How we look is central and body perception is influenced by the appearance ideals that exist in society. Goffman says that the body´s appearance is socialized, shaped and transformed to fit into the society in which it is created. The appearance and creation are visible to the individual and to other people, thereby influencing the identity. The roles are presented to an audience of the same or similar type in which the body is of great importance because it shows to the world who you are (Goffman, 1959). The men in this study strove to fill the role of the perfect male ideal to display what they had achieved.

For the men in this study, the acquired perfect male body included aspects that are essential to existence. According to existential philosophy, a human being does not choose her/his existence, but by being aware of her/his choices, he/she can choose how he/she wants to live and create meaning in her/his life (Heidegger, 1927/1998; Sartre, 1986). In our study, bodies were used as instruments to achieve goals and to receive confirmation. This improved the men´s self-confidence and raised their self-esteem. The meaning of self-identity is an entire unite that is intertwined with meanings of self-confidence, self-affirmation, self-image. This means that self-identity is inseparable of all meanings that relate to the “self”. It creates the person.

Sartre (1986) states that we constantly are on our way away from being (that which is just now) and striving for nothingness (our dreams and goals). For these men to be able to achieve their dreams and goals and not being judged for their choices, they needed to lie. They did not want to be exposed to the feeling of not being genuine and thus jeopardize the social role they had achieved. According to existentialism, the individual is responsible for the conscious choices he/she makes and therefore he/she is able to decide over her/his own life. However, there is also a constant struggle between different choices that makes us anxious (Sartre, 1986). Anxiety is constant because the choices we make affect others. This may lead to problems in both work and private life. The choice of using AAS as men in this study did, might have led to body failure. The context of meaning may change when something with which we handle the world breaks down, such as a tool, in this case the body (Heidegger, 1927/1998). Existential free choices are limited in the event of illness. Pressure from relatives influenced the men in their choice of continuing to use AAS. They experienced this as jeopardizing their very existence. Having to quit one´s lifestyle may be experienced as having to leave one´s body physically, mentally and socially. It may feel like the end of life. Existence is what frightens us when we are left to ourselves (Sartre, 1986).

AAS are illegal (Riksdagen, 1992) and therefore AAS users are a hard-to-reach population. It is easier to recruit men than women because the use is much more common in male populations. A total of twelve interviews were included in this study. We used a phenomenological research approach with a lifeworld perspective, so despite few interviews, data collection has contributed with meanings (Polit & Beck, 2017) forming the basis for an analysis and generating an essential description of the phenomenon: men´s use of AAS. The results entail a development of knowledge to be able to understand men using AAS.

Descriptions have been based on the men’s lived experiences and there is of course a risk that the informants may have chosen their answers taking the illegality of AAS into account. We, however, believe that in-depth interviews with support for reflection have contributed to a truthful material since substantive meanings have emerged. One weakness of the method is that it is time-consuming and requires knowledge in interview techniques. In order to gain a deeper understanding, in-depth interviews as a method have been shown to be a strength.

## Conclusions and clinical implications

Men´s use of AAS is a complex phenomenon. It partly concerns a traditional view of masculinity that is reflected in society. It also involves an individual driving force to achieve a body ideal that has winning characteristics, not only in competitions. This driving force to attract attention and to be the best in the group blinds users to the negative consequences of using AAS, which do not become visible to these men until illness, injury or other dysfunction arises. Bodybuilding seems to be based on low self-esteem and a competitive instinct among men. However, the built-up body is fragile from both an existential and a biological perspective. Self-esteem and self-confidence can quickly be destroyed in the absence of confirmation from others and the side effects of the substances may damage the body in the form of serious physiological disorders. Therefore, both broad and deep knowledge and understanding are required to be able to meet these men in their problems and vulnerability, a meeting which is hampered by their low trust in healthcare, and by the fact that the AAS are illegal.
